# Clinical Efficacy, Safety, and Immunogenicity of a Live Attenuated Tetravalent Dengue Vaccine (CYD-TDV) in Children: A Systematic Review with Meta-analysis

**DOI:** 10.3389/fimmu.2017.00863

**Published:** 2017-08-04

**Authors:** Moffat Malisheni, Svetlana F. Khaiboullina, Albert A. Rizvanov, Noah Takah, Grant Murewanhema, Matthew Bates

**Affiliations:** ^1^Ministry of Health, Lusaka, Zambia; ^2^Institute of Health and Wellbeing, University of Glasgow, Glasgow, United Kingdom; ^3^Department of Microbiology and Immunology, National University of Singapore, Singapore, Singapore; ^4^Institute of Molecular and Cell Biology, Agency for Science, Technology and Research, Singapore, Singapore; ^5^Department of Microbiology and Immunology, University of Nevada Reno, Reno, NV, United States; ^6^Kazan Federal University, Kazan, Russia; ^7^Ministry of Health, Yaounde, Cameroon; ^8^College of Health Sciences, University of Zimbabwe, Harare, Zimbabwe; ^9^University College London Research & Training Programme, University of Zambia, University Teaching Hospital, Lusaka, Zambia; ^10^HerpeZ, University Teaching Hospital, Lusaka, Zambia

**Keywords:** dengue hemorrhagic fever, dengue shock syndrome, CYD-TDV, dengue virus, efficacy, safety, immunogenicity

## Abstract

**Background:**

Dengue hemorrhagic fever is the leading cause of hospitalization and death in children living in Asia and Latin America. There is an urgent need for an effective and safe dengue vaccine to reduce morbidity and mortality in this high-risk population given the lack of dengue specific treatment at present. This review aims to determine the efficacy, safety, and immunogenicity of CYD-TDV vaccine in children.

**Methods:**

This is a systematic review including meta-analysis of randomized controlled clinical trial data from Embase, Medline, the Cochrane Library, Web of Science, and ClinicalTrials.gov. Studies that assessed CYD-TDV vaccine efficacy [(1 − RR)*100], safety (RR), and immunogenicity (weighted mean difference) in children were included in this study. Random effects model was employed to analyze patient-level data extracted from primary studies.

**Results:**

The overall efficacy of CYD-TDV vaccine was 54% (40–64), while serotype-specific efficacy was 77% (66–85) for DENV4, 75% (65–82) for DENV3, 50% (36–61) for DENV1, and 34% (14–49) for DENV2. 15% (−174–74) vaccine efficacy was obtained for the unknown serotype. Meta-analysis of included studies with longer follow-up time (25 months) revealed that CYD-TDV vaccine significantly increased the risk of injection site reactions (RR = 1.1: 1.04–1.17; *p*-value = 0.001). Immunogenicity (expressed as geometric mean titers) in descending order was 439.7 (331.7–547.7), 323 (247 – 398.7), 144.1 (117.9–170.2), and 105 (88.7–122.8) for DENV3, DENV2, DENV1, and DENV4, respectively.

**Conclusion:**

CYD-TDV vaccine is effective and immunogenic in children overall. Reduced efficacy of CYD-TDV vaccine against DENV2 notoriously known for causing severe dengue infection and dengue outbreaks cause for serious concern. *Post hoc* meta-analysis of long-term follow-up data (≥25 months) from children previously vaccinated with CYD-TDV vaccine is needed to make a conclusion regarding CYD-TDV vaccine safety in children. However, CYD-TDV vaccine should be considered for use in regions where DENV2 is not endemic as currently there is no specific treatment for dengue infection.

## Introduction

Continuously increasing dengue virus (DENV) related morbidity and mortality poses a serious threat to global public health and has exerted pressure on national health budgets of endemic countries. There are four types of genetically distinct dengue viruses (DENV 1–4) (1), all causing severe dengue infection (2, 3). Brady et al. estimates that four billion people are at risk of acquiring dengue infection worldwide (4) with approximately 284–528 million dengue cases being documented annually (5). Dengue hemorrhagic fever (DHF)/Dengue Shock Syndrome (DSS) comprise 500,000 to one million of these cases leading to over 20,000 fatalities mostly in children (6, 7). The goal of World Health Organization is to reduce dengue related morbidity and mortality by 2020 (8). Despite the availability of vector control programs, dengue infection has continued to rise globally (9) with significant economic burdens and might continue to do so in the future given the ongoing climate change. Introducing an efficacious and safe vaccine in endemic regions has the potential to reduce dengue related hospitalization and death in children due to severe dengue infection (DHF/DSS). The risk of developing DHF/DSS during secondary infection is increased when an individual is exposed to a dengue serotype that is different from the one previously experienced (10). This occurs due to antibody-dependent enhancement (ADE) which involves low levels of cross-reactive neutralizing antibodies produced during primary dengue infection forming complexes with target cell receptors (3, 11, 12). Consequently enhancing the number of dengue-infected cells and viremia (3, 11, 12). Therefore, a tetravalent dengue vaccine capable of eliciting a balanced immune response against all four dengue serotypes is warranted if complications due to ADE are to be averted (11, 13, 14). CYD-TDV vaccine is the most advanced live attenuated tetravalent dengue vaccine (15). However, comprehensive evidence regarding CYD-TDV vaccine efficacy, safety, and immunogenicity in children exclusively is absent.

A meta-analysis of randomized controlled trials (RCTs) by da Costa et al. has demonstrated that CYD-TDV vaccine is safe and induces a balanced immune response (16). However, safety and immunogenicity were determined for all age groups and no subgroup analysis based on the age of included participants was conducted. The fact that dengue infection is more severe in children compared to adults indicates that the two groups might respond to CYD-TDV vaccine differently with a possibility of adults confounding the true effect of the vaccine in children. The study also assessed vaccine efficacy by combining five primary studies. However, only Sabchareon et al. out of the five combined studies was designed to determine CYD-TDV vaccine efficacy in Thai children (17). The determined vaccine efficacy was 30.2% and was not statistically significant. Two large Phase III clinical studies designed to determine CYD-TDV vaccine efficacy and not included in the meta-analysis by da Costa et al. have been conducted in Asian and Latin American children showing efficacy of 56.5 and 60.8%, respectively (9, 18). However, because these studies were conducted in various age groups from different regions, the findings are not directly comparable. Therefore, to comprehensively address all the aforementioned concerns, we decided to determine the efficacy, safety, and immunogenicity of CYD-TDV vaccine in children by answering the following questions: (i) does CYD-TDV vaccine reduce the incidence of virologically confirmed dengue (VCD) cases in vaccinated compared with unvaccinated children? (ii) Does CYD-TDV vaccine increase the risk of adverse events in vaccinated as compared to the unvaccinated children? (iii) Is there a difference in geometric mean titers (GMTs) between children exposed to CYD-TDV vaccine and those unexposed?

## Methods

### Eligibility Criteria and Definitions

This review was conducted and reported in accordance with the Cochrane and preferred reporting items for systematic reviews and meta-analyses guidelines (19, 20). Population: children were defined as all individuals under the age of 18 years (21). Intervention: CYD-TDV vaccine manufactured by Sanofi Pasteur. Vaccine was reconstituted in 0.4% sodium chloride and 2.5% serum albumin. Comparator: standard of care, placebo, or no intervention. Outcome: the primary end assessing points were CYD-TDV vaccine efficacy in accordance with the “Guidelines for clinical trials of dengue vaccine in endemic areas” (22). Reduction in the incidence of VCD cases per protocol analysis. Safety: AEs—unfavorable medical occurrences that are not treatment related and ARs—those that might be treatment related (23). Immunogenicity: levels of dengue neutralizing antibodies expressed as GMTs and measured using plaque reduction neutralisation test with a 50% plaque reduction threshold (PRNT50). More information on the PRNT50 test can be obtained in the “guidelines for plaque reduction neutralization testing of human antibodies to dengue viruses” (24). Study design: only RCTs were included in this review. CYD-TDV vaccination interval requirement was that immunizations be conducted at months 0, 6, and 12 (three vaccine regimen). Exclusion criteria: studies which did not assess CYD-TDV vaccine efficacy, safety, or immunogenicity or did not use CYD-TDV vaccine; studies involving participants aged over 17 years; and studies that used non-RCT study designs, non-three vaccine regimen or a three vaccine regimen with a different vaccination interval.

### Literature Search and Data Extraction

A comprehensive search strategy was developed in collaboration with an experienced medical librarian to identify recently published studies as presented in Appendix I (all referenced appendices are in the Supplementary Material). Embase, Medline, Web of Science, the Cochrane Library, ClinicalTrials.gov, references of included studies, and authors served as sources for published data. Gray literature was not searched because it lacks quality control. The search for published data was initiated on 01/03/2016, concluded on 11/05/2016 and pilot tested according to the method proposed by Long (25) (Appendix II, Figure 1 in Supplementary Material). This was done to ensure that the analysis includes all relevant information and fit to achieve the goal of this study. Corresponding authors of primary studies were contacted to request for numerical data or clarifications in cases where data were incomplete or graphically presented (Appendix II, Figure 2 in Supplementary Material). Information was extracted based on individual patient-level data.

### Data Items and Summary Measures

Primary end points and their respective summary measures included: overall and serotype-specific CYD-TDV vaccine efficacy (per protocol analysis), CYD-TDV safety [immediate AEs, severe adverse events (SAEs), solicited ARs, solicited injection site reactions (pain, erythema, and swelling), solicited systemic reactions (fever, headache, malaise, myalgia, and asthenia) and unsolicited adverse event (UAEs)]. Relative risk (RR) was the preferred summary measure for CYD-TDV efficacy [(1 − RR)*100] (17) and safety, whereas immunogenicity (measured as GMTs) was estimated using the weighted mean difference (WMD). Relative risk was defined as the ratio of incidence VCD cases in the CYD-TDV vaccine group divided by the ratio of incidence VCD cases in the unvaccinated group (26). Mean difference was defined as an absolute difference between the GMTs in the intervention and control groups.

### Risk of Bias and Statistical Analysis

The Cochrane Handbook for Systematic Reviews of Interventions tool (27) was used in this analysis to assess the quality of each of the included studies. The risk of bias was assessed both at the study and the outcome levels. Evidence tables served as the starting point for data synthesis. The tables were reviewed as well as the forest plots to determine the possibility of combining data from studies in a meta-analysis. The *I*^2^ and *Q* statistics were used to formally check for the presence of heterogeneity and consequently determine whether the effect sizes should be pooled. Heterogeneity was classified as low, medium and high for *I*^2^ values corresponding to 25, 50, and 75%, respectively (28). If heterogeneity was either low/medium or reduced to these levels after being resolved, the pooled effect sizes of outcomes were explored. However, if variations in the effect size between pooled studies remained high (*I*^2^ ≥ 75%) after efforts to resolve heterogeneity, meta-analysis was not conducted. The heterogeneity was investigated using meta-regression and subgroup analysis to explain its possible cause. The Cochrane Collaboration recommends that studies with divergent effect sizes from the rest should be excluded to resolve heterogeneity (27). The influence of individual studies on the overall effect size was formally investigated using meta-influence. Identified studies were removed systematically to reduce heterogeneity across the combined studies. Sensitivity analysis was performed to explore the robustness of the findings using the fixed effects model (Appendices III and IV in Supplementary Material). The random effects model was preferred because the true effect size may not be constant across all the included studies (29) given that they were conducted in different age groups, countries, regions, and ethnic groups. Extracted data were exported from Excel spreadsheet to STATA version 13.0, where all statistical analyses were conducted. Publication bias was explored by employing Harbord’s and Egger’s tests.

## Results

**Figure 1:**
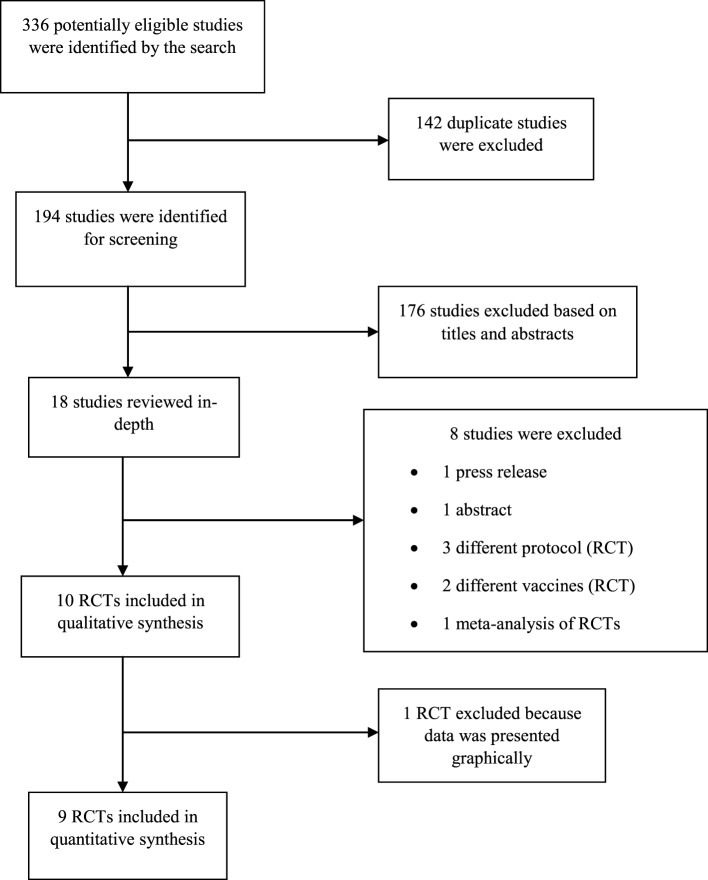
Study selection process.

336 articles were selected for this study. Embase, Medline, Web of Science, the Cochrane Library, and ClinicalTrials.gov yielded 104, 80, 53, 46, and 53 articles, respectively (Figure 1 below). Duplicate studies were removed using Mendeley reference manager leaving 194 published articles for further analysis. Of these, 174 articles were removed based on titles and abstracts. After an in-depth review, additional eight articles were excluded, including a press release (30), an abstract (31), one paper used a two regimen vaccination protocol (32), two papers used different vaccines, Acambis (33) and PVRV (34), and one meta-analysis of RCTs previously mentioned (16). Also, two studies utilizing a different protocol of the vaccination regimen and vaccine reconstitution protocol were excluded (35, 36). At the end of the selection procedure, nine studies were found eligible for inclusion in meta-analysis (9, 17, 18, 37–42) after one study was excluded (43) because it presented all data graphically.

A summary of the included studies’ characteristics is presented in Table 1. Additional study activities are presented in Appendix II, Table 1 in Supplementary Material. All three studies that assessed vaccine efficacy used reverse transcription polymerase chain reaction and enzyme-linked immunosorbent assay tests to confirm dengue cases. Six studies did not specify how information regarding CYD-TDV vaccine safety was collected, while four requested parents/guardians of participants to record safety profiles. PRNT50 was utilized by all included studies to determine immunogenicity. Details regarding the aforesaid are summarized in Appendix II, Table 2 in Supplementary Material. Quality assessment of eligible studies is presented in Table 2. Regarding RR and WMD analyses the values of no difference were 1 and 0, respectively. Therefore, 95% confidence intervals (95% CI) that traversed 1 regarding the former and 0 the latter were considered statistically insignificant (alpha > 0.05). This review considered three hypotheses: CYD-TDV vaccination does not reduce the incidence of VCD cases in children; CYD-TDV vaccination increases the risk of ARs in children; and there is no difference in GMT levels between vaccinated and unvaccinated children. Variables used in meta-regression and subgroup analyses included: gender, sample size, randomization ratio, blinding method, placebo type, age group, study location, RCT phase, and flavivirus (DENV, yellow fever, and Japanese encephalitis) seroprevalence at baseline.

**Table 1:** Study characteristics of the vaccine trials that meet the inclusion criteria.

Reference	Study design (phase)	Sample size		Age range (years)	Males		Outcome determined	Seroprevalence at baseline	
		CYD-TDV	Placebo		CYD-TDV (%)	Placebo (%)		CYD-TDV	Placebo
Crevat et al. (43)	RCT (II)	60	30	1–1.25	63	60	Safety and immunogenicity	DENV or JE; 45%	DENV or JE; 50%
Villar et al. (9)	RCT (III)	12,574	6,261	9–16	49.7	49.6	Efficacy and safety	DENV; 80%	DENV; 77%
Capeding et al. (18)	RCT (III)	6,851	3,424	2–14	48	48	Efficacy and safety	DENV or JE; 79%	DENV or JE; 77%
Amar-Singh et al. (40)	RCT (III)	199	51	2–11	48	62.7	Safety and immunogenicity	FV-positive; 44%	
								FV-negative; 35%	
Villar et al. (41)	RCT (II)	401	199	9–16	49.1	45.7	Safety and immunogenicity	FV; 78.8%	FV; 80.8%
Dayan et al. (37)	RCT (II)	100	50	9–16	40	55	Safety and immunogenicity	DENV or YF; 81%	DENV or YF; 84%
Leo et al. (38)	RCT (II)	898	300	2–17	44.0	54.4	Safety and immunogenicity	Not stated	Not stated
Tran et al. (39)	RCT (II)	120	60	2–17	48	58	Safety and immunogenicity	DENV or JE; 71%	DENV or JE; 78%
Sabchareon et al. (17)	RCT (IIb)	2,669	1,333	4–11	48	48	Efficacy, safety, and immunogenicity	DENV or JE; 91%	DENV or JE; 92%
Lanata et al. (42)	RCT (II)	199	99	2–11	51	46	Safety and immunogenicity	DENV; 37%	DENV; 48%

*RCT, randomized controlled trial; DENV, dengue virus; YF, yellow fever; JE, Japanese encephalitis; FV, flavivirus*.

**Table 2:** Risk of bias assessment.

Reference	Selection bias		Blinding		Attrition	Reporting bias	Other bias	Researcher’s judgment
	Randomization	Concealment	Performance	Detection				
Crevat et al. (43)	Low	Unknown	Low	Unknown	Low	Low	Unknown	Unknown
Villar et al. (9)	Low	Low	Low	Low	Low	Low	Unknown	Low
Capeding et al. (18)	Low	Low	Low	Low	Low	Low	Unknown	Low
Amar-Singh et al. (40)	High	Low	High	Low	Low	Low	Unknown	High
Villar et al. (41)	Low	Low	High	Low	Low	Low	Unknown	High
Dayan et al. (37)	Low	Low	High	Low	Low	Low	Unknown	High
Leo et al. (38)	Low	Low	High	Low	Low	Low	Unknown	High
Tran et al. (39)	Low	Low	High	Low	Low	Low	Unknown	High
Sabchareon et al. (17)	Low	Low	High	Low	Low	Low	Unknown	High
Lanata et al. (42)	Low	Low	Low	Low	Low	Low	Unknown	Low

**Risk of bias**	**Interpretation**		**Within trial bias**			**Across trial bias**		

Low	Bias, if present, is unlikely to alter the results seriously		Low risk of bias for all key domains			Most information is from trials at low risk of bias		
Unknown	A risk of bias that raises some doubt about the results		Low or unclear risk of bias for all key domains			Most information is from trials at low or unclear risk of bias		
High	Bias may alter the results seriously		High risk of bias for one or more key domains			The proportion of information from trials at high risk of bias is sufficient to affect the interpretation of results		

### CYD-TDV Vaccine Efficacy

The review found a statistically significant pooled overall CYD-TDV vaccine efficacy of 54% (40–64; *p*-value < 0.001). This result implies that the vaccine reduces the risk of acquiring dengue infection in the intervention group relative to the control group by 54%. Serotype-specific efficacy showed that CYD-TDV vaccine was more effective against DENV4 (77%: 66–85; *p*-value < 0.001) and DENV3 (75%: 65–82; *p*-value < 0.001), while it was less effective against DENV1 (50%: 36–61; *p*-value < 0.001), DENV2 (34%: 14–49; *p*-value = 0.002) and unknown DENV serotype (15%: −174–74; *p*-value = 0.79). There was convincing evidence to reject the pre-specified null hypothesis for all but the unknown serotype, which was not statistically significant. The main findings for CYD-TDV efficacy including evidence for the presence of heterogeneity and publication bias are summarized in Table 3.

**Table 3:** Main CYD-TDV efficacy findings.

CYD-TDV efficacy	Number of studies pooled	Intervention *n*/*N*	Control *n*/*N*	Heterogeneity (*p*-value)	Harbord’s test for publication bias (*p*-value)	RR (95% CI)	Efficacy = (RR − 1)*100 (95% CI)	*p*-Value
Overall	3	338/21,736	386/10,832	61.6% (0.074)	0.396	0.46 (0.36–0.60)	54% (40–64)	<0.001
DENV1	3	126/21,736	126/10,832	0.0% (0.97)	0.482	0.50 (0.39–0.64)	50% (36–61)	<0.001
DENV2	3	127/21,736	96/10,832	0.0% (0.45)	0.359	0.66 (0.51–0.86)	34% (14–49)	0.002
DENV3	3	54/21,736	107/10,832	0.0% (0.91)	0.062	0.25 (0.18–0.35)	75% (65–82)	<0.001
DENV4	3	35/21,736	78/10,832	0.0% (0.61)	NA	0.23 (0.15–0.34)	77% (66–85)	<0.001
Unknown serotype	3	12/21,736	6/10,832	17.3% (0.3)	NA	0.85 (0.26–2.74)	15% (−174–74)	0.79

*N, sample size; n, number of cases recorded; NA, not applicable (could not be calculated because input data contained 0 values); DENV 1–4, dengue virus serotypes*.

### CYD-TDV Vaccine Safety

Generally, solicited injection site reactions (any) and solicited systemic reactions (any, fever, headache, and asthenia) showed an increased but statistically insignificant risk in vaccinated children compared with unvaccinated children. Other than the aforesaid, CYD-TDV vaccine reduced the risk of ARs. However, meta-analysis of included studies with longer follow-up time (25 months) revealed that CYD-TDV vaccination increased the risk of solicited injection site reactions; RR = 1.1 (1.04–1.17; *p*-value = 0.001) and RR = 1.09 (0.97–1.22; *p*-value = 0.145) using the fixed and random effects models, respectively. However, only the fixed model showed a statistically significant risk (Appendix IV, Figure 8 in Supplementary Material). Insignificantly increased risk of solicited systemic reactions was also observed using both the random and fixed effects models (Appendix IV, Figures 10 and 11 in Supplementary Material). None of the aforementioned variables used in subgroup analyses were associated with finding of a statistically significant heterogeneity (*I*^2^ > 90%; *p*-value < 0.001). However, meta-regression analysis demonstrated that gender explained 95.1% (*p*-value = 0.049) and 96.5% (*p*-value = 0.002) of the variability in the studies that assessed solicited reactions and solicited injection site reactions, respectively (Appendix IV, Figures 2 and 3 in Supplementary Material). Negative gender coefficients entailed that for every unit increase in the proportion of males in the CYD-TDV group, the log RR reduced by 0.45 and 0.91 units for solicited reactions and injection site reactions, respectively. Following stratification of solicited reactions and injection site reactions by gender, heterogeneity in the subgroups of the pooled studies with more males plummeted to 0.0% (*p*-value < 0.41) and 61.4% (*p*-value = 0.035), respectively (Table 4). A study by Crevat et al. found that the most frequently reported safety profiles in the intervention group were UAEs (60–70%), solicited ARs (50–55%), solicited systemic reactions (40–50%), and solicited injection site reactions (<20%) (43). There were no immediate AEs or SAEs reported.

**Table 4:** Main CYD-TDV safety findings.

Safety profile	Number of studies combined	Intervention *n*/*N*	Control *n*/*N*	Heterogeneity (*p*-value)	Harbord’s test for publication bias (*p*-value)	RR (95% CI)	*p*-Value
SAE	7	783/24,304	447/12,020	0.0% (0.51)	0.52	0.86 (0.77–0.96)	0.009
Solicited reactions^a^	2	133/299	110/149	0.0% (0.41)	NA	0.64 (0.55–0.74)	<0.001
UAE	7	1,709/4,239	847/2,003	18.7% (0.29)	0.02	0.95 (0.88–1.03)	0.19
**Solicited injection site reactions occurring between day 0 and 7 post vaccination**							
Any^a^	5	2,112/3,935	898/1,848	61.4% (0.035)	0.64	1.06 (0.97–1.16)	0.2
Pain (any)^a^	3	151/663	191/329	52.2% (0.12)	0.48	0.40 (0.30–0.51)	<0.001
Erythema (any)	5	72/1,140	50/504	67.1% (0.016)	0.42	0.55 (0.27–1.11)	0.09
Swelling (any)	5	50/1,140	41/504	67.2% (0.016)	0.37	0.47 (0.20–1.06)	0.07
**Solicited systemic reaction occurring between day 0 and 14 post vaccination**							
Any	7	2,694/4,234	1,266/1,998	33.9% (0.17)	0.52	1.0 (0.95–1.06)	0.9
Fever (any)	5	187/1,138	75/504	48.1% (0.1)	0.94	1.08 (0.76–1.53)	0.67
Headache (any)	5	401/1,140	173/504	60.8% (0.03)	0.44	1.0 (0.78–1.3)	0.97
Malaise (any)	5	314/1,140	133/504	55.9% (0.059)	0.96	0.99 (0.75–1.32)	0.96
Myalgia (any)	5	312/1,140	158/504	68.0% (0.014)	0.788	0.8 (0.59–1.08)	0.15
Asthenia (any)	5	177/1,140	69/504	56.8% (0.055)	0.093	1.14 (0.75–1.74)	0.55

*N, sample size; n, number of cases recorded; NA, not applicable (could not be calculated because few studies were pooled); SAE, severe adverse events; UAE, unsolicited adverse events*.

*^a^All parameters were determined after resolving heterogeneity by excluding divergent studies*.

### CYD-TDV Vaccine Induced Immunogenicity

Although it was challenging to determine the overall immunogenicity using the random effects model due to the persistence of variation in the effect sizes even after resolving serotype-specific heterogeneity, the fixed effects model generated 74.28 1/dil (69.90–78.68; *p*-value < 0.001). The combined serotype-specific GMT levels found after resolving heterogeneity in descending order was: DENV3 (439.7 1/dil), DENV2 (323 1/dil), DENV1 (144.1 1/dil), and DENV4 (105.71 1/dil). A different order was detected when the fixed effects model was applied; DENV3 (114.56 1/dil), DENV4 (112.34 1/dil), DENV2 (81.91 1/dil), and DENV1 (40.51 1/dil) (Appendix IV in Supplementary Material). This showed that the estimates of immunogenicity were not robust. Subgroup analysis and the systematic elimination of studies with divergent mean differences revealed that combining studies with different ages resulted in significant heterogeneity, except for DENV1. This was observed even within the same study (Appendix IV, Figures 4 and 5 in Supplementary Material). By contrast, pooling together studies with the same age resulted in low heterogeneity (*I*^2^ = 0.0%), except for DENV4 (Appendix IV, Figures 6 and 7 in Supplementary Material). The main immunogenicity findings and respective 95% CIs are summarized in Table 5. The highest neutralizing antibody titers in descending order reported by Cravat et al. were as follows: DENV3 (311–387 1/dil), DENV2 (147–213 1/dil), DENV4 (127–160 1/dil), and DENV1 (105–124 1/dil) (43). Tran et al. reported the following GMTs: DENV1—4:129, 216, 169, and 146 1/dil, respectively (39). The study further demonstrated that GMTs in children increased with increasing age: 2–5 years (64.7–143 1/dil), 6–11 years (93.9–185 1/dil) and 12–17 years (135–334 1/dil).

**Table 5:** Main CYD-TDV immunogenicity findings.

Dengue serotype	Number of studies combined	Heterogeneity (*p*-value)	Egger’s test for publication bias (*p*-value)	WMD expressed as GMTs (95% CI)	*p*-Value
DENV1	6	93.8% (<0.001)	0.01	107.5 (70.1–144.9)	<0.001
DENV1^a^	5	11% (0.34)	0.11	144.1 (117.9–170.2)	<0.001
DENV2	6	95.4% (<0.001)	0.007	176.9 (115.4–238.4)	<0.001
DENV2^a^	3	14.9% (0.31)	0.53	323.1 (247–398.7)	<0.001
DENV3	6	95.2% (<0.001)	0.013	221.9 (152.6–291.2)	<0.001
DENV3^a^	3	41.6% (0.18)	0.5	439.7 (331.7–547.7)	<0.001
DENV4	6	94.4% (<0.001)	0.04	152.9 (110.6–195.3)	<0.001
DENV4^a^	3	37.4% (0.2)	0.65	105.7 (88.7–122.8)	<0.001

*WMD, weighted mean difference expressed as GMTs; GMTs, geometric mean titers (1/dil); DENV 1–4, dengue virus serotypes*.

*^a^All parameters were determined after resolving heterogeneity by excluding divergent studies*.

## Discussion

### CYD-TDV Vaccine Efficacy

Although our findings show that CYD-TDV vaccine is protective against dengue infection overall, its reduced efficacy against DENV2 is extremely worrying. This is because DENV2 is known to cause severe dengue infection and is twice as likely to result in DHF/DSS compared to other serotypes (6). The Asian DENV2 has also been reported to cause outbreaks of DHF/DSS, highly pathogenic and is gradually replacing the less pathogenic Latin American variant (44). All efficacy studies conducted in Asia demonstrated reduced and statistically insignificant vaccine efficacy against DENV2 compared with the one conducted in Latin America. This might indicate that CYD-TDV vaccine induced antibodies readily neutralize the less pathogenic Latin American than the highly virulent Asian DENV2 variant. It has been reported that DENV2 neutralizing antibodies induced after primary infection with DENV1 demonstrate differential neutralizing activity against the Asian and Latin American DENV2 variants (45).

It is worth noting that the observed 32% CYD-TDV vaccine protection against DENV2 is merely the best estimate. The real vaccine efficacy in the population can be as low as 14% (Table 3). Another important point to note is that CYD-TDV vaccine induced DENV2 neutralizing antibodies had the second highest GMT levels and yet provided the least protection. The question is why? Villar et al. concluded that GMT levels elicited by CYD-TDV vaccine do not reflect serotype-specific vaccine efficacy (9). It has also been suggested that dengue antibodies either might not be the immunological correlate of protection or that each dengue serotype has its own protective titer threshold (46). Both findings correspond with our results which show that CYD-TDV vaccine protection was highest for the serotype with the lowest GMT levels and so on (Tables 3 and 5). Microevolution due to genetic recombination and natural selection occurring within individual dengue serotypes might explain lower efficacy of the CYD-TDV vaccine against DENV2 (47–49). Wahala et al. have shown that mutations occurring in the E protein (the major target for dengue neutralizing antibodies) have an effect on antibody binding and neutralizing activity (50). Therefore, reduced vaccine efficacy against DENV2 could be due to the fact that the circulating DENV2 virus acquired mutations in the E protein hence becoming antigenically and genetically different from the one included in the CYD-TDV vaccine. Reduced DENV2 vaccine efficacy might as well be as a result of dominant CYD-TDV vaccine induced antibodies that lack or have low serotype-specific neutralizing activity against the circulating DENV2 serotype. This is because high levels of serotype-specific neutralizing antibodies are known to confer protection against subsequent DENV infection (45). Evidence depicting a similar situation can be derived from the seasonal influenza vaccine which was only 23% effective in vaccinees that were infected with a subtype that was different from the one included in the vaccine (51). Although information regarding the unknown DENV serotype is limited, a study by Mustafa et al. has proposed the emergence of a DENV5 serotype (48). By contrast, Hesse argues that the aforesaid is highly unlikely because DENV mutation rate is too low to lead to the creation of a new serotype (44).

### CYD-TDV Vaccine Safety

Our overall findings regarding the safety of CYD-TDV vaccine are in agreement with the findings by da Costa et al. (16) in that none of the increased AEs and ARs were statistically significant. Crevat et al. also concluded that CYD-TDV vaccine does not increase the risk of AEs and ARs in children below 2 years (43). However, this study had a small sample size (*N* = 90), short follow-up time (18 months), and it is not clear how allocation concealment was done. All of which might lead to the actual effect of CYD-TDV vaccine being overestimated. Contrary to the aforesaid, revelations from meta-analysis of studies with longer follow-up time indicate that CYD-TDV increases the risk of injection site reactions significantly thus making it difficult to reject the pre-specified hypothesis (Appendix IV, Figure 8 in Supplementary Material). Similarly, *post hoc* analysis of the data from Capeding et al. (18) has demonstrated that the risk of hospitalization and severe dengue in children aged between 2 and 5 years vaccinated with CYD-TDV was highly significant (RR = 7.45: 1.15–313.8, *p*-value not provided) (52). However, *post hoc* meta-analysis of all studies conducted to assess CYD-TDV vaccine safety in children is required before a comprehensive conclusion can be made.

### CYD-TDV Vaccine Induced Immunogenicity

Our immunogenicity findings show that GMTs significantly increased in CYD-TDV vaccinated compared to unvaccinated children. Children in Latin America had higher GMT levels compared to those in Asia. Da Costa et al. (16) have reported similar results and evidence from our findings is sufficient to reject the pre-specified null hypothesis. However, there was significant variation in the effect sizes presented in the included studies. Interestingly, reduced heterogeneity was observed when studies with the same age group were combined regardless of study location except for DENV4. By contrast, combining studies with different age groups resulted in significantly increased heterogeneity regardless of study location and, surprisingly, even within the same study. Furthermore, we found that GMT effect sizes increased as the age of study participants increased, which corresponds with the findings by Tran et al. (39). Taken together, our findings clearly demonstrate that age influences CYD-TDV vaccine induced GMT levels. Included studies demonstrated that prior exposure to dengue viruses increased antibody response during subsequent infection. The observed variations in GMT levels among the included studies might be explained by variations in the burden of dengue infection across countries and between the two regions, with Asian countries experiencing a higher burden compared with Latin American countries (53). Although the PRNT is considered the gold standard, discrepancies between laboratories and regions have been reported (54, 55). Furthermore, the scientific community has different views regarding this test. Rainwater-Lovett et al. and Thomas et al. have separately reported that PRNT gives varying results based on the test conditions applied (56, 57). Endy has described as confusing the fact that PRNT does not give information on whether an individual will be protected from subsequent dengue infection using cross-reactive neutralizing antibodies (58). The former concern might be responsible for the observed variations in CYD-TDV vaccine elicited GMT levels across the included studies, while the later may be more applicable to the reduced vaccine efficacy against DENV2 as stated above. To the contrary, Timiryasova et al. have concluded that the PRNT test is fit for purpose and can “detect and measure dengue serotype-specific neutralizing antibodies in human serum samples with acceptable intra-assay and inter-assay precision, accuracy/dilutability, specificity, and with a lower limit of quantitation of 10” (59).

### Strengths and Limitations

This, to the best of our knowledge, is the first systematic review to assess CYD-TDV vaccine efficacy not only in children exclusively but also using primary studies that were designed specifically to determine vaccine efficacy. Our review has demonstrated that children of varying ages respond to CYD-TDV vaccination differently and gender imbalance in the CYD-TDV group introduces heterogeneity when assessing solicited reactions and injection site reactions. Excluding graphically presented data prevented estimation bias. Since all of the included studies were conducted in children located in Asia and Latin America, the findings cannot be generalized to children of all endemic regions.

### Implications for Public Health and Research

Emergence of novel and virulent dengue serotypes has been proposed. To prevent possible pandemics, there is need to strengthen vector control programs, dengue surveillance, diagnostic capabilities and management, and research through capacity building in endemic settings such as Asia, Latin America, and especially Africa, where dengue infection happens to be neglected (60, 61). DENV2 included in the CYD-TDV vaccine needs to be updated to match the currently circulating Asian and Latin American variants. In addition, understanding of dengue neutralizing antibodies by investigating whether they are correlates of protection and what titer thresholds against all four serotypes are optimal for protection is warranted. Therefore, the scientific community needs to quickly come to a consensus regarding the PRNT test. Otherwise, new serological tests capable of being standardized to enable inter laboratory comparability, reproducibility and that can accurately and specifically measure dengue correlates of protection must be developed given that this is crucial for vaccine development. The fact that dengue infection is known to cause DHF/DSS and death in children is an indication that passive immunotherapy using serotype-specific neutralizing monoclonal antibodies that target conserved regions of the E protein might be a viable alternative to vaccination. Passive immunotherapy might significantly benefit younger children who are at higher risk and yet respond poorly to vaccines immunologically. Another reason for considering passive immunotherapy is that higher titers of serotype-specific neutralizing monoclonal antibodies can be administered without enhancing ADE, which is mainly caused by low titer levels of cross-reactive antibodies as previously mentioned. Future vaccine trials should consider employing ≥25 months follow-up time, stratify and provide age specific data to facilitate comprehensive and conclusive analyses. Finally, *post hoc* analysis can reveal vital vaccine-related safety information missed during the duration of the clinical trial. Therefore, the aforementioned analysis should be considered and encouraged where complete clinical data of participants involved in clinical trials are available.

## Conclusion

Overall, CYD-TDV vaccine is effective, but less efficacious against DENV2 in children. CYD-TDV vaccine is immunogenic in children with lower GMT levels observed in younger children compared to adolescents. Although the vaccine increased the risk of some safety parameters in vaccinated children insignificantly, meta-analysis of studies with long follow-up time revealed that CYD-TDV vaccine significantly increased the risk of solicited injection site reactions. Therefore, *post hoc* meta-analysis of the long term follow-up data (≥25 months) collected from the children previously vaccinated with CYD-TDV are needed before a comprehensive conclusion regarding CYD-TDV vaccine safety in children can be made. However, given the urgency for a dengue vaccine in endemic regions, CYD-TDV should be considered for use in regions where DENV2 is not endemic as currently there is no specific treatment for dengue infection.

## Author Contributions

MM designed the study, performed the statistical analysis, and wrote the final report. SK, NT, and GM independently performed searches, selection, and data extraction of published articles. AR resolved disagreements from searches, selection, and data extraction. MB resolved disagreements from searches, selection, and data extraction. All authors made contributions and reviewed the final report.

## Conflict of Interest Statement

The authors declare that the research was conducted in the absence of any commercial or financial relationships that could be construed as a potential conflict of interest.

## Abbreviations

ADE, antibody-dependent enhancement; AE, adverse events; AR, adverse reactions; CYD-TDV, live-tetravalent dengue vaccine; DENV, dengue virus; DHF, dengue hemorrhagic fever; DSS, dengue shock syndrome; ELISA, enzyme-linked immunosorbent assay; GMT, geometric mean titre; JE, Japanese encephalitis; PRISMA, preferred reporting items for systematic reviews and meta-analyses; PRNT50, plaque reduction neutralisation test with a 50% plaque reduction threshold; RCT, randomized controlled trial; RR, relative risk; RT-PCR, reverse transcription polymerase chain reaction; SAE, severe adverse events; UAE, unsolicited adverse events; UN, United Nations; VCD, virologically confirmed dengue; WHO, World Health Organization; WMD, weighted mean difference; YF, yellow fever.
