# Supplementary material for: Clinical Efficacy, Safety, and Immunogenicity of a Live Attenuated Tetravalent Dengue Vaccine (CYD-TDV) in Children: A Systematic Review with Meta-analysis
Source: Front Immunol. 2017 Aug 4;8:863. doi: 10.3389/fimmu.2017.00863 (PMC5543029; doi:10.3389/fimmu.2017.00863)
Supplement: Supplementary file 1 [file Data_Sheet_1.PDF]

Supplementary information

Appendix I: Search strategy
Database: Embase 1947-Present, updated daily
Search Strategy:
----
1 vaccin*.mp. (428734)
2 dengue vaccine.tw. (595)
3 tetravalent dengue vaccine.tw. (180)
4 tetravalent vaccine.tw. (186)
5 recombinant tetravalent dengue vaccine.tw. (9)
6 recombinant tetravalent vaccine.tw. (1)
7 CYD-TDV.tw. (38)
8 TDV.tw. (194)
9 prevention/ (219500)
10 (prophylact* or prophylax*).mp. [mp=title, abstract, heading word, drug trade name, original title, device manufacturer, drug manufacturer, device trade name, keyword] (262899)
11 protection/ (61059)
12 Live attenuated Tetravalent Dengue Vaccine.tw. (61)
13 Live-attenuated Tetravalent Dengue Vaccine.tw. (61)
14 Live Attenuated Tetravalent Vaccine.tw. (10)
15 or/1-14 (903861)
16 safety.tw. (508498)
17 allergic reaction/ (25990)
18 hyperergic reaction/ (44476)
19 hypersensitive response/ (25990)
20 hypersensitiveness/ (44476)
21 adverse reaction/ (176411)
22 adverse event$/ (27)
23 adverse effect$/ (176411)
24 side effect$/ (216916)
25 immune reaction/ (70382)
26 metabolic side effects/ (176411)
27 reactogenicity.tw. (1928)
28 reactogenecity.tw. (8)
29 or/16-28 (980345)
30 immunogenecity.tw. (238)
31 immunogenicity.tw. (35391)
32 immune response/ (191681)
33 immune provocation/ (0)
34 immune activation/ (53)
35 immune induction/ (0)
36 host response/ (191681)
37 antibody production/ (72635)
38 antibody generation/ (0)
39 antibody synthesis/ (72635)
40 immunologic$ enhancement/ (25559)
41 or/30-40 (303730)
42 ((clinical adj efficacy) or efficacy).tw. (816775)
43 ((clinical adj effectiveness) or effectiveness).tw. (422002)
44 (potent or potency).tw. (429826)
45 dengue.tw. (18498)
46 DENV/ (8605)
47 dengue disease/ (0)
48 dengue fever/ (15116)
49 dengue virus/ (8605)
50 dengue infection/ (0)
51 dengue flavivirus/ (8605)
52 dengue haemorrhagic fever/ (15116)

53 dengue haemorrhagic fever/ (15116)
54 dengue shock syndrome/ (7347)
55 classic$ dengue/ (0)
56 break bone fever/ (0)
57 break-bone fever/ (0)
58 breakbone fever/ (15116)
59 or/42-44 (1573163)
60 or/45-58 (21340)
61 29 or 41 or 59 (2513893)
62 15 and 60 and 61 (1366)
63 limit 62 to human (942)
64 limit 62 to animals (265)
65 63 and 64 (0)
66 64 not 65 (265)
67 62 not 66 (1101)
68 clinical trial/ (866297)
69 randomized controlled trial/ (403226)
70 randomization/ (70526)
71 single blind procedure/ (22086)
72 double blind procedure/ (132853)
73 crossover procedure/ (47302)
74 placebo/ (291281)
75 randomi?ed controlled trial$.tw. (135034)
76 rct.tw. (20268)
77 random allocation.tw. (1596)
78 randomly allocated.tw. (24890)
79 allocated randomly.tw. (2138)
80 (allocated adj2 random).tw. (909)
81 single blind$.tw. (17644)
82 double blind$.tw. (171379)
83 ((treble or triple) adj blind$).tw. (590)
84 placebo$.tw. (241595)
85 prospective study.tw. (151084)
86 exp clinical trial/ (1093093)
87 exp research design/ (4276887)
88 or/68-87 (5083261)
89 case study/ (46914)
90 case report.tw. (330619)
91 Abstract report/ or letter/ (977441)
92 comparative study/ (722601)
93 exp evaluation studies/ (32310)
94 (control$ or prospectiv$ or volunteer$).tw. (4590330)
95 or/89-94 (6485207)
96 88 and 95 (1690679)
97 95 not 96 (4794528)
98 67 and 97 (203)
99 67 not 98 (898)
100 limit 99 to (infant <to one year> or child <unspecified age> or preschool child <1 to 6 years> or school child <7 to 12 years> or adolescent <13 to 17 years>) (104)
101 limit 99 to (meta analysis or "systematic review") (8)

Appendix II

Table 1. Evidence table

Author, Year	Country of study	Follow-up (months)	Study design	Randomisation Ratio	Intervention	Control	Nunber of doses (month administered)
CrevatD et al., 2015	Philippines	18	Phase II	2:1	CYD-TDV	Varicella (OKAVAX; Sanofi Pasteur, France) and two hepatitis-A (AVAXIM 80; Sanofi Pasteur, France)	3 (0, 6 and 12)
VillarL et al., 2014	Brazil<br>Colombia<br>Honduras<br>Mexico<br>Puerto Rico	25	Phase III	2:1	CYD-TDV	Placebo; 0.9 NaCl	3 (0, 6 and 12)
CapedingMR et al., 2014	Malaysia<br>Philippines<br>Thailand<br>Vietnam	25	Phase III	2:1	CYD-TDV	Placebo; 0.9 NaCl	3 (0, 6 and 12)
Amar-Singh HSS et al., 2013	Malaysia	18	Phase III	4:1	CYD-TDV	Placebo; 0.9 NaCl	3 (0, 6 and 12)
VillarL et al., 2013	Colombia<br>Honduras<br>Mexico<br>Puerto Rico	18	Phase II	2:1	CYD-TDV	Placebo; 0.9 NaCl	3 (0, 6 and 12)
Dayan GH et al., 2013	Brazil	18	Phase II	2:1	CYD-TDV	Placebo; 0.9 NaCl	3 (0, 6 and 12)
Leo YS et al., 2012	Singapore	18	Phase II	3:1	CYD-TDV	Placebo; 0.9 NaCl	3 (0, 6 and 12)
Tran HN et al., 2012	Vietnam	18	Phase II	2:1	CYD-TDV	Placebo; 0.9 NaCl	3 (0, 6 and 12)
Sabchareon A et al., 2012	Thailand	25	Phase IIb	2:1	CYD-TDV	Inactivated rabies vaccine for the first injection of the first 50 children randomly assigned to the control group, and 0.9% NaCl saline placebo for all other injections.	3 (0, 6 and 12)
LanataCF et al., 2012	Peru		Phase II	2:1	CYD-TDV	Placebo; 0.9 NaCl, pneumococcal polysaccharide vaccine (PPS) at month 12	3 (0, 6 and 12)

Table 2. Summary of data collection and analysis

Author, Year	Efficacy		Safety		Immunogenicity	
	Confirmatory test	Analysis	Data collection	Analysis	Serological test	Analysis
Crevat et al., 2015*			From parents/guardians	Included participants who received ≥ 1 injections	PRNT50	Included participants who received ≥ 1 injection
Villar et al., 2014	RT-PCR and ELISA	Incidence DENV cases to estimate efficacy	From parents/guardians	-	PRNT50	-
Capeding MR et al., 2014	RT-PCR and ELISA	Incidence DENV cases to estimate efficacy	-	Included participants who received ≥ 1 injections	PRNT50	-
Amar-Singh et al., 2013*			-	Included participants who received ≥ 1 injections	PRNT50	Included participants who received ≥ 1 injection
Villar et al., 2013*			-	-	PRNT50	-
Dayan et al., 2013*			-	Included participants who received ≥ 1 injections	PRNT50	Included participants who received ≥ 1 injection
Leo et al., 2012*			From parents/guardians	Included participants who received ≥ 1 injection	PRNT50	Included participants who received ≥ 1 injection
Tran et al., 2012*			Parents/guardians	Included participants who received ≥ 1 injection	PRNT50	Included participants who received ≥ 1 injection
Sabchareon et al., 2012	RT-PCR and ELISA	Incidence DENV cases to estimate efficacy	-	-	PRNT50	-
Lanata et al., 2012*			-	Included participants who received ≥ 1 injection	PRNT50	Included participants who received ≥ 1 injection

(*) studies did not assess CYD-TDV vaccine efficacy; (-) not specified

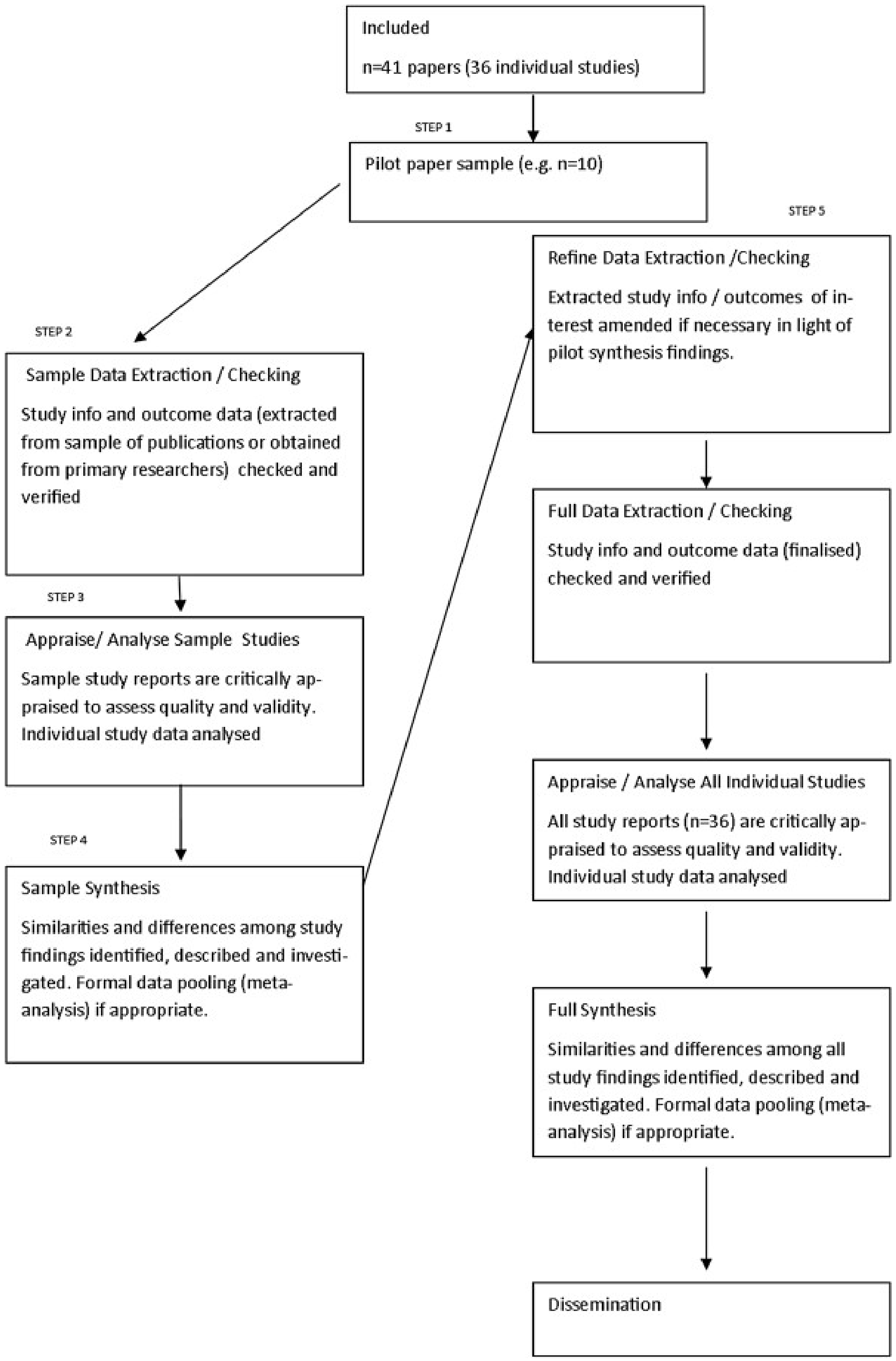

Figure 1. Pilot method

Studies that assessed efficacy			Date of contact		
			17th - 20th June, 2016		
**Author, Year**	**Age in years**	**Note**	**Email Response**	**Sent**	**Email address**
Villar et al., 2014	9 to 16				
Capeding et al., 2014	2 to 14				
Sabchareon et al., 2012	4 to 11				
da Costa et al., 2014	2 to 45	Request for efficacy data	Delivery to the following recipient failed permanently: mlmoreli@jatai.ufg.br	Yes	mlmoreli@jatai.ufg.br
**Studies that assessed safety**					
**Author, Year**	**Age in years**	**Note**			
Crevat et al., 2015	1 to 1.25	Data presented graphically	I am not working any longer within Sanofi Pasteur and do not have the requested data . I forward your message to my colleague Dr Thelma Laot who managed the trial locally in close collaboration with Dr Capeding. She should be able to provide you with the data.	Yes	dcnp0169@gmail.com
Villar et al., 2014	9 to 16				
Capeding et al., 2014	2 to 14				
Amar-Singh et al., 2013	2 to 11				
Villar et al., 2013	9 to 16				
Dayan et al., 2013	9 to 16				
Leo et al., 2012	2 to 45				
Tran et al., 2012	2 to 45				
Sabchareon et al., 2012	4 to 11				
Lanata et al., 2012	2 to 11				
Capeding et al., 2011	2 to 45	Data presented graphically	Not delivered. The e-mail address you entered couldn't be found.	Yes	denis.crevat@sanofipasteur.com
Poo et al., 2011	2 to 45	Data presented graphically	Not delivered. The e-mail address you entered couldn't be found.	Yes	Gustavo.dayan@sanofipasteur.com
**Studies that assessed immunogenicity**					
**Author, Year**	**Age in years**	**Note**			
Crevat et al., 2015	1 to 1.25	Data presented graphically	I am not working any longer within Sanofi Pasteur and do not have the requested data . I forward your message to my colleague Dr Thelma Laot who managed the trial locally in close collaboration with Dr Capeding. She should be able to provide you with the data.	Yes	dcnp0169@gmail.com
Amar-Singh et al., 2013	2 to 11				
Villar et al., 2013	9 to 16				
Dayan et al., 2013	9 to 16				
Leo et al., 2012	2 to 45				
Tran et al., 2012	2 to 45	Data presented graphically	Pending	Yes	wartel-tram.anh@sanofipasteur.com
Sabchareon et al., 2012	4 to 11				
Lanata et al., 2012	2 to 11				
Capeding et al., 2011	2 to 45	Data presented graphically	Not delivered. The e-mail address you entered couldn't be found.	Yes	denis.crevat@sanofipasteur.com
Poo et al., 2011	2 to 45	Data presented graphically	Not delivered. The e-mail address you entered couldn't be found.	Yes	Gustavo.dayan@sanofipasteur.com

Figure 2. Corresponding author contact

Risk of bias summery figure (adopted from the Cochrane Collaboration)

Author, Year	Random sequence generation (selection bias)	Allocation concealment (selection bias)	Blinding of participants and researchers (performance bias)	Blinding of outcome assessment (detection bias)	Incomplete outcome data (attrition bias)	Selective reporting (reporting bias)	Other bias
Crevat et al., 2015	Low risk	Unknown risk	Low risk	Unknown risk	Low risk	Low risk	Unknown risk
Villar et al., 2014	Low risk	Low risk	Low risk	Low risk	Low risk	Unknown risk	Unknown risk
Capeding et al., 2014	Low risk	Low risk	Low risk	Low risk	Low risk	Unknown risk	Unknown risk
Amar-Singh et al., 2013	High risk	Low risk	High risk	Low risk	Low risk	Low risk	Unknown risk
Villar et al., 2013	Low risk	Low risk	High risk	Low risk	Low risk	Low risk	Unknown risk
Dayan et al., 2013	Low risk	Low risk	High risk	Low risk	Low risk	Low risk	Unknown risk
Leo et al., 2012	Low risk	Low risk	High risk	Low risk	Low risk	Low risk	Unknown risk
Tran et al., 2012	Low risk	Low risk	High risk	Low risk	Low risk	Low risk	Unknown risk
Sabchareon et al., 2012	Low risk	Low risk	High risk	Low risk	Low risk	Low risk	Unknown risk
Lanata et al., 2012	Low risk	Low risk	Low risk	Low risk	Low risk	Low risk	Unknown risk
Capeding et al., 2011	Unknown risk	Unknown risk	High risk	Low risk	Low risk	Low risk	Unknown risk
Poo et al., 2011	Unknown risk	Unknown risk	High risk	Low risk	Low risk	Low risk	Unknown risk

KEY

✓	Low risk
!	Unknown risk
✗	High risk

Figure 3. Risk of bias of included studies

Appendix III

Results: tables

Table 1. Pooled RRs and respective p-values from fixed effects model (CYD-TDV safety).

Safety profile	RR (95% CI)	p-value
Severe adverse events	0.86 (0.77 – 0.97)	0.01
Solicited reactions	0.87 (0.80 – 0.94)	< 0.001
Unsolicited adverse events	0.95 (0.89 – 1.01)	0.125
Solicited injection site reactions occurring between day zero and seven post vaccination.		
Any	1.04 (0.98 – 1.09)	0.194
Pain (any)	0.58 (0.52 – 0.65)	< 0.001
Erythema (any)	0.59 (0.42 – 0.85)	0.004
Swelling (any)	0.51 (0.34 – 0.77)	0.001
Solicited systemic reaction occurring between day zero and 14 post vaccination.		
Any	1.0 (0.96 – 1.04)	0.88
Fever (any)	1.09 (0.86 – 1.40)	0.48
Headache (any)	1.01 (0.87 – 1.16)	0.95
Malaise (any)	0.99 (0.83 – 1.18)	0.92
Myalgia (any)	0.83 (0.70 – 0.97)	0.02
Asthenia (any)	1.12 (0.87 – 1.45)	0.38

Table 2. Pooled WMDs and respective p-values from fixed effects model (CYD-TDV immunogenicity).

Dengue serotype	WMD expressed as GMTs (95% CI)	p-value
Overall	74.28 (69.90 – 78.67)	< 0.001
DENV1	40.51 (34.01 – 47.02)	< 0.001
DENV2	81.91 (71.86 – 91.95)	< 0.001
DENV3	114.56 (102.39 – 126.73)	< 0.001
DENV4	112.34 (103.14 – 121.53)	< 0.001

WMD = weighted mean difference expressed as GMTs; GMTs = geometric mean titres (1/dil); DENV1 – 4 = dengue virus serotypes

Appendix IV

Results: figures

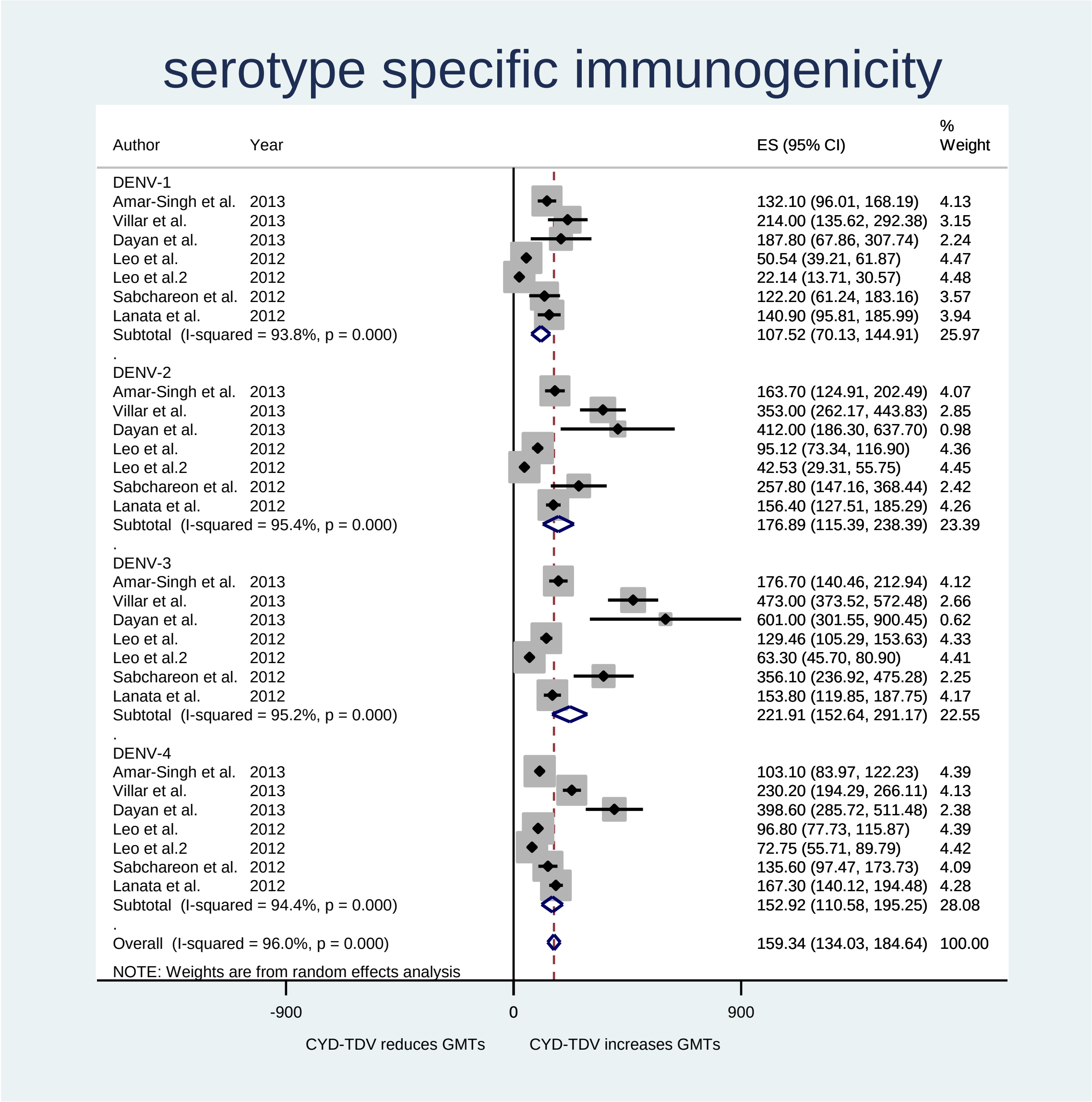

Figure 1. Forest plot showing serotype specific immunogenicity [ES = effect size (WMD)]

```
Meta-regression                                       Number of obs   =        4
REML estimate of between-study variance               tau2            = .003487
% residual variation due to heterogeneity             I-squared_res   =  37.35%
Proportion of between-study variance explained        Adj R-squared   =  95.05%
With Knapp-Hartung modification
```

logRR	Coef.	Std. Err.	t	P>|t|	[95% Conf.	Interval]
gender	-.4511784	.1038545	-4.34	0.049	-.8980282	-.0043286
_cons	1.804796	.4485646	4.02	0.057	-.1252216	3.734813

Figure 2. Meta-regression (solicited reaction)

```
Meta-regression                                      Number of obs  =        7
REML estimate of between-study variance              tau2           = .005863
% residual variation due to heterogeneity            I-squared_res  =   58.22%
Proportion of between-study variance explained       Adj R-squared  =   96.45%
With Knapp-Hartung modification
```

logRR	Coef.	Std. Err.	t	P>|t|	[95% Conf. Interval]	
gender	-.9087209	.1501158	-6.05	0.002	-1.294606	-.5228358
_cons	3.692875	.6160378	5.99	0.002	2.109299	5.27645

Figure 3. Meta-regression (solicited injection site reaction)

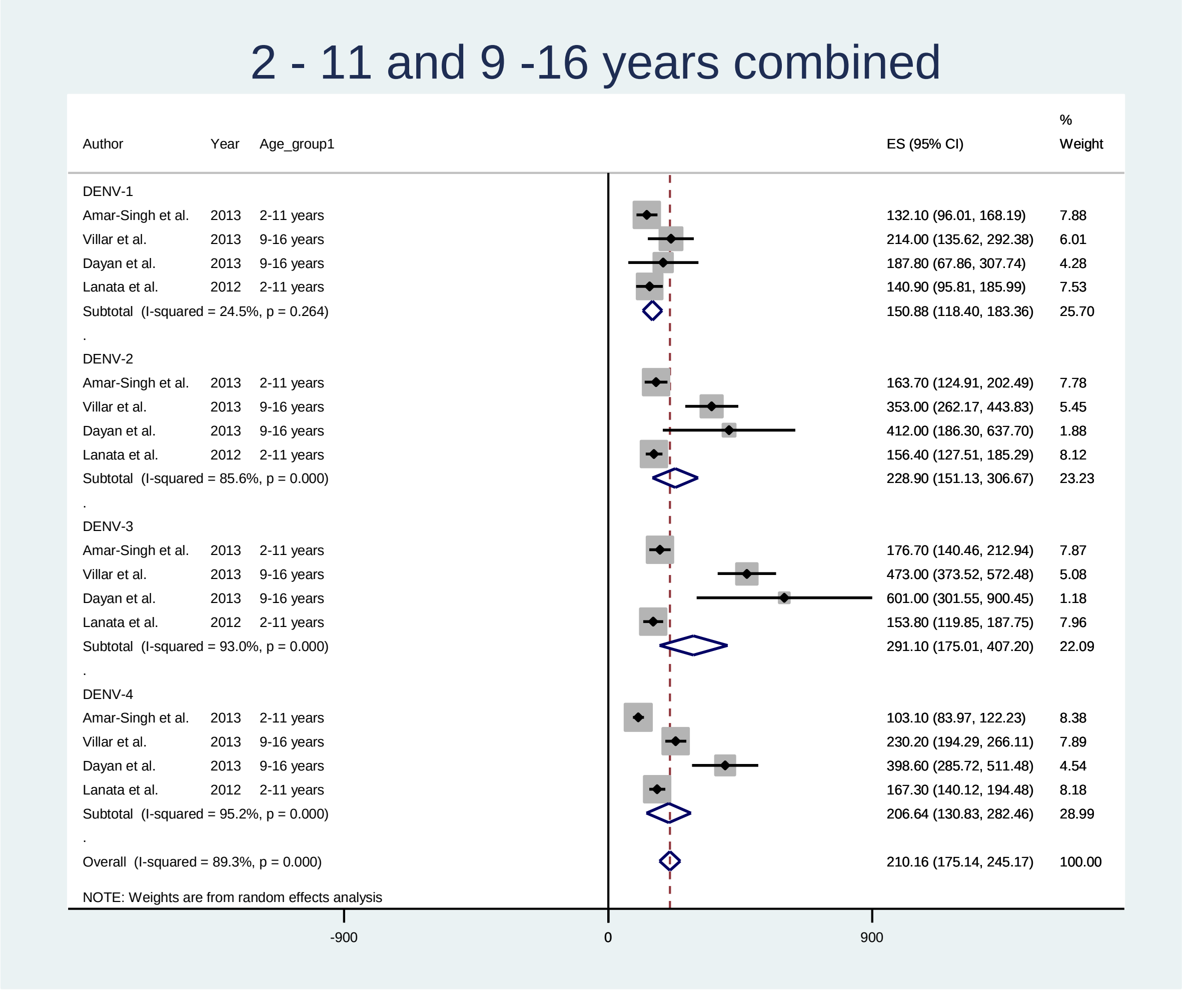

Figure 4. Forest plot demonstrating persistence of heterogeneity [ES = effect size (WMD)]

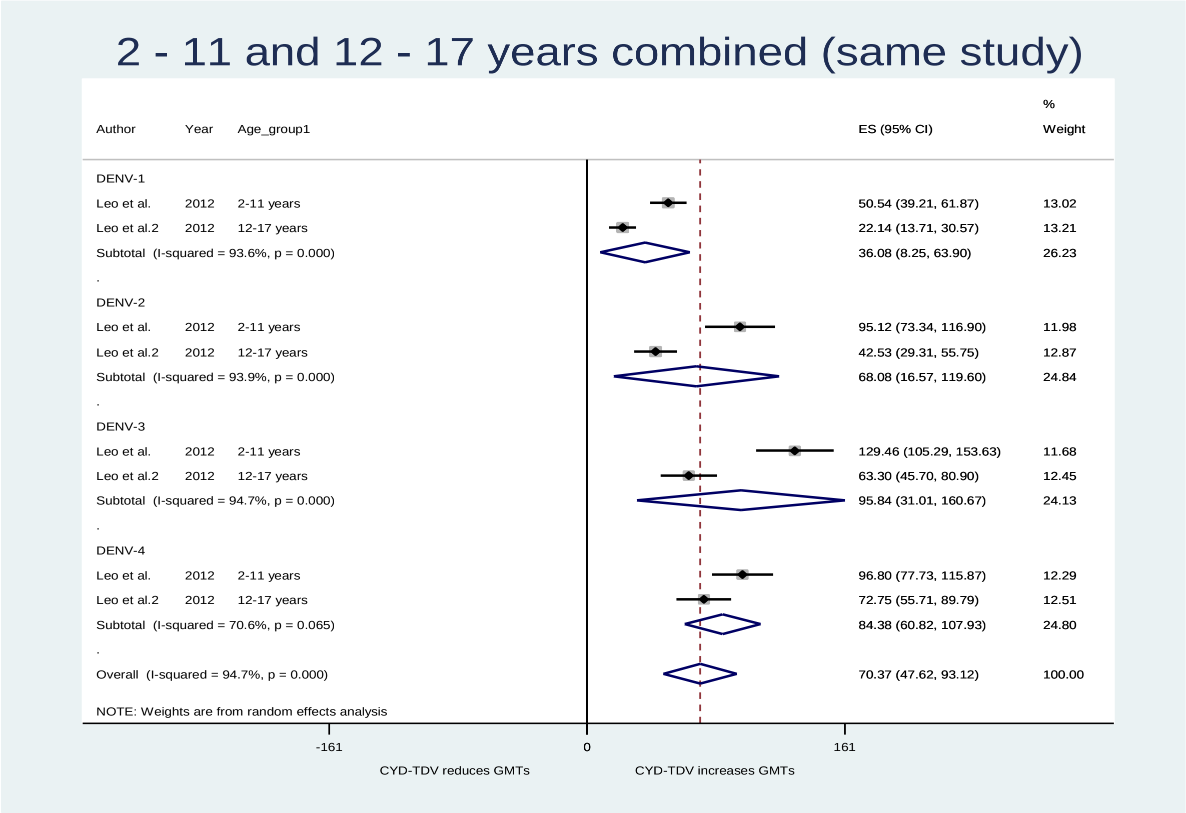

Figure 5. Forest plot demonstrating persistence of heterogeneity [ES = effect size (WMD)]

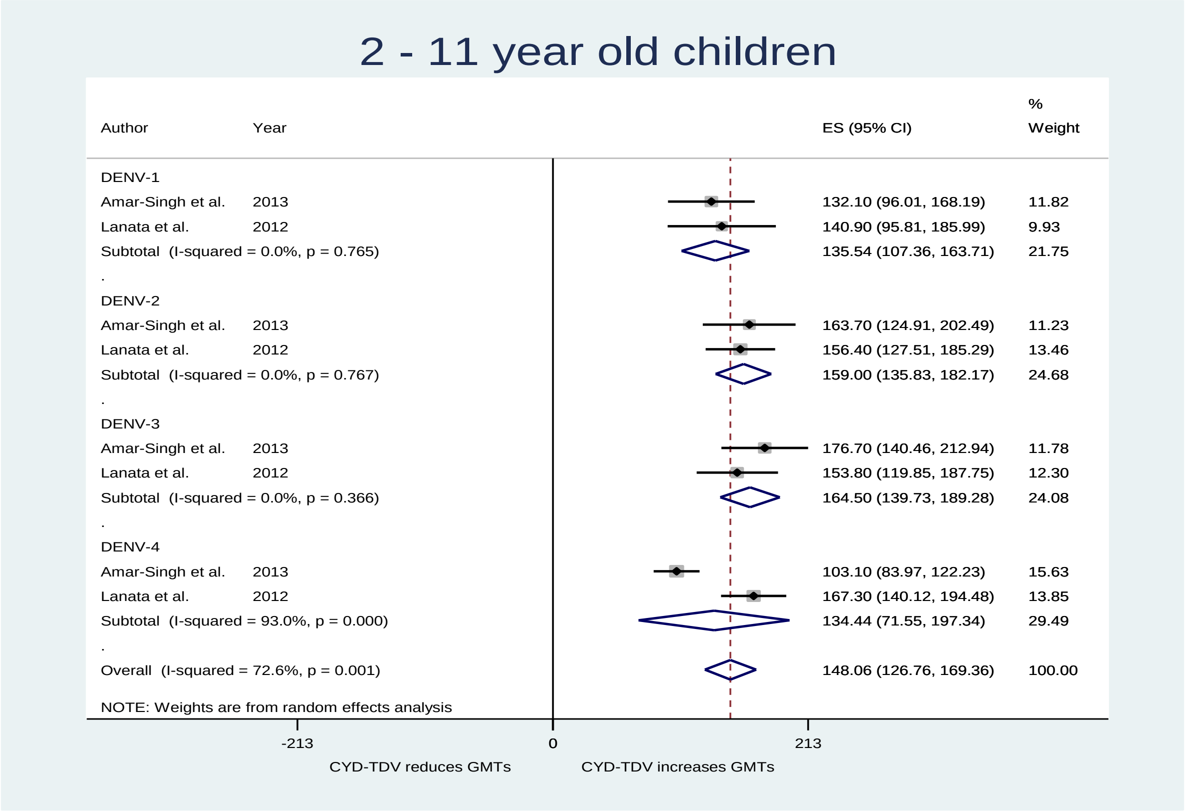

Figure 6. Forest plot demonstrating persistence of heterogeneity [ES = effect size (WMD)]

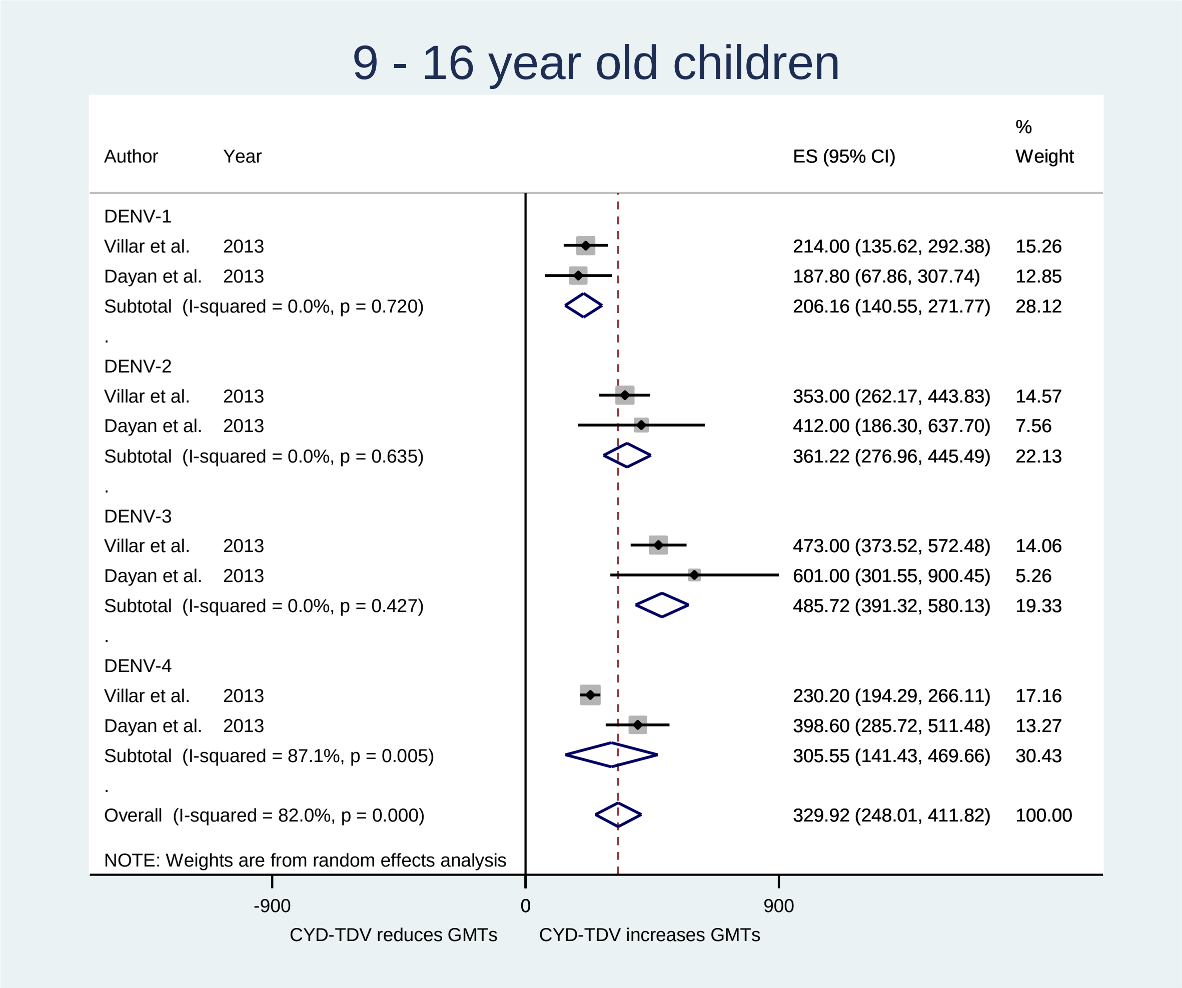

Figure 7. Forest plot demonstrating persistence of heterogeneity [ES = effect size (WMD)]

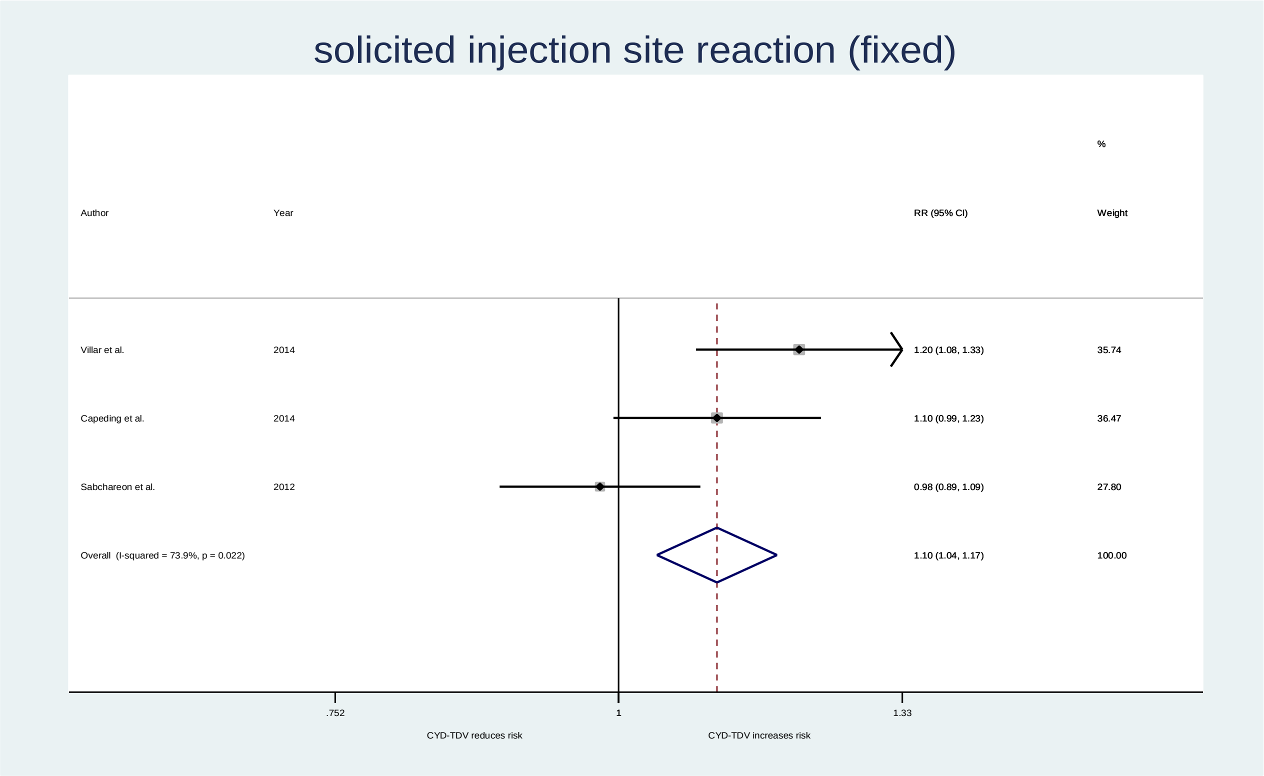

Figure 8. Forest plots showing pooled effect sizes from studies with 25 months follow-up time

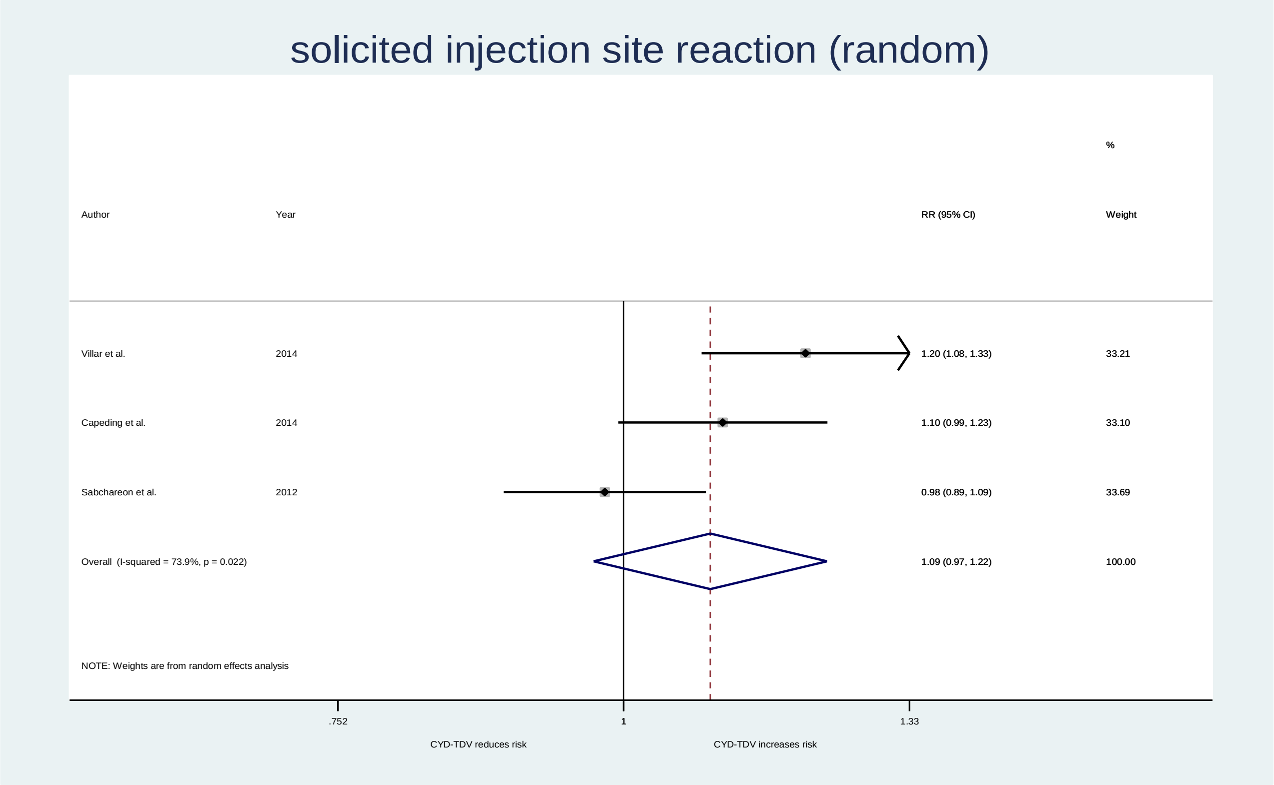

Figure 9. Forest plots showing pooled effect sizes from studies with 25 months follow-up time

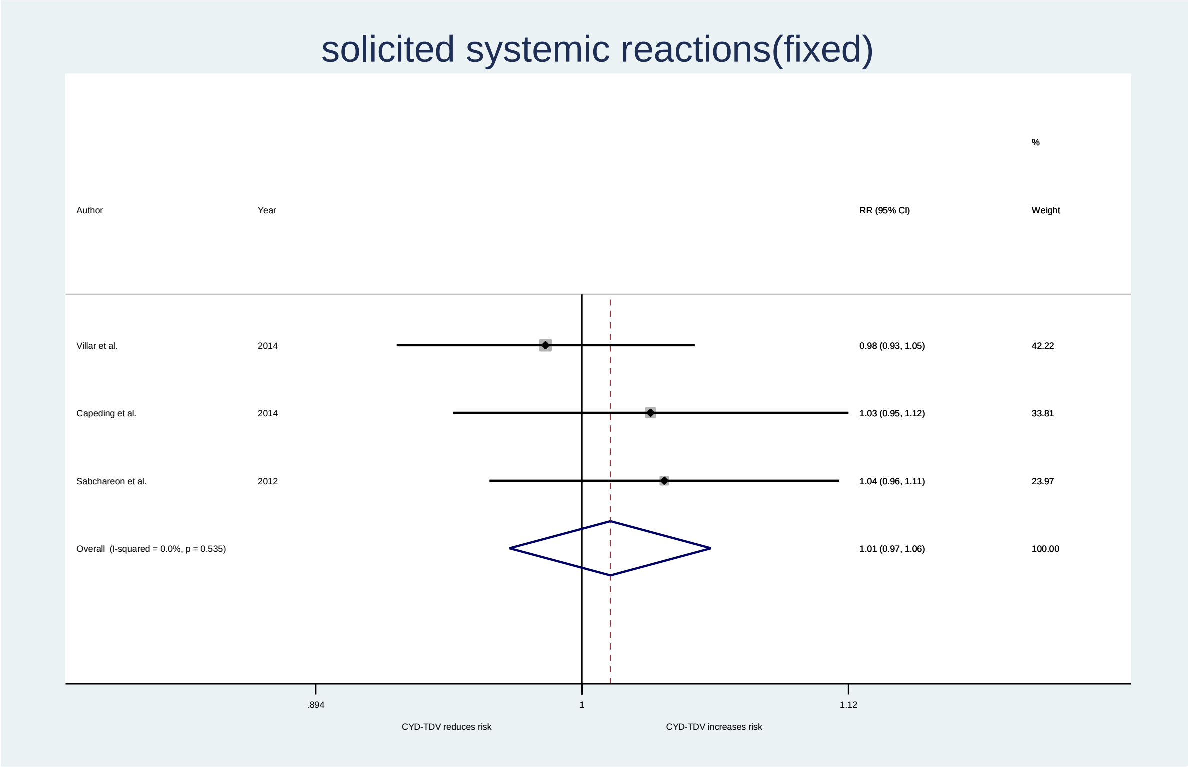

Figure 10. Forest plots showing pooled effect sizes from studies with 25 months follow-up time

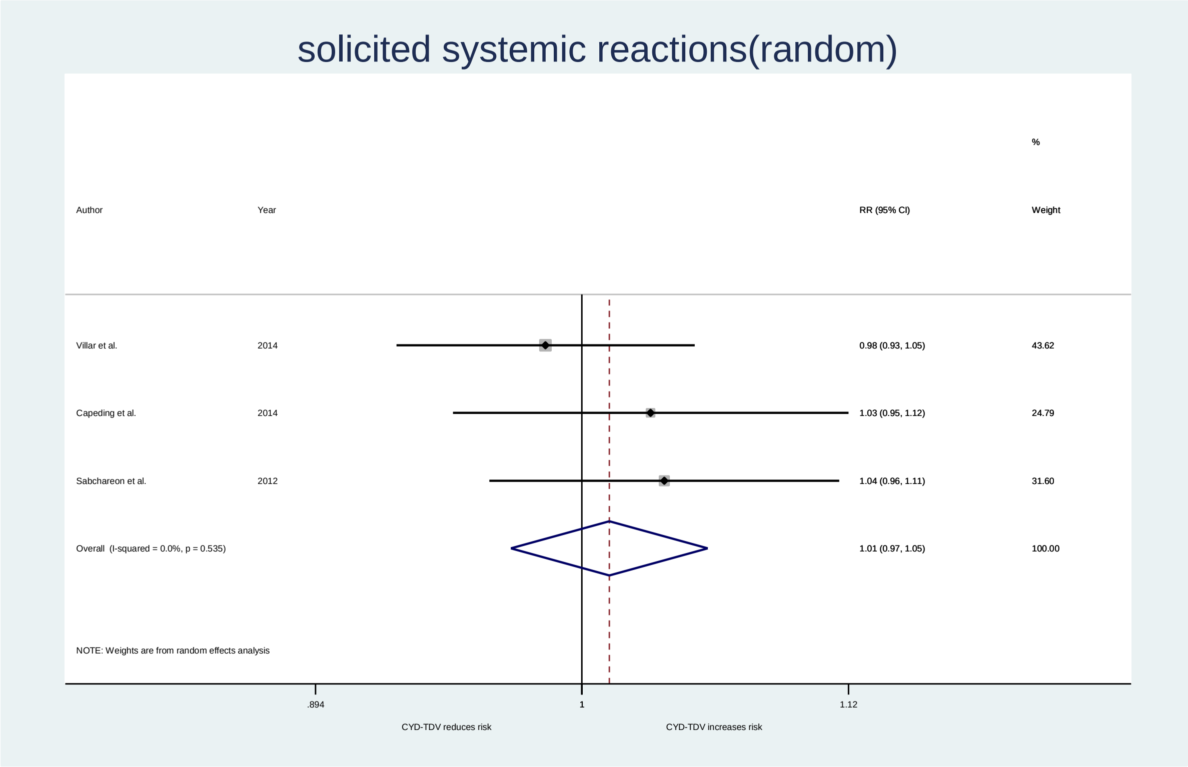

Figure 11. Forest plots showing pooled effect sizes from studies with 25 months follow-up time
